# Optimization of Vitamin B1, B2, and B6 Absorption in Nang Tay Dum Floating Rice Grains

**DOI:** 10.3390/foods13172650

**Published:** 2024-08-23

**Authors:** Thi Thao Loan Nguyen, Thi Mong Nghi Pham, Thanh Binh Ho, Binh Ly-Nguyen

**Affiliations:** 1 Institute of Food and Biotechnology, Can Tho University, Can Tho City 900000, Vietnam; thloankt@yahoo.com (T.T.L.N.); nghib1908735@student.ctu.edu.vn (T.M.N.P.); 2 Faculty of Chemical and Food Technology, Ho Chi Minh City Industry and Trade College, Ho Chi Minh City 700000, Vietnam; 3 Faculty of Agriculture and Natural Resources, An Giang University, Vietnam National University, Ho Chi Minh City, Long Xuyen 880000, Vietnam; htbinh@agu.edu.vn

**Keywords:** absorption, coating, floating rice, Nang Tay Dum rice variety, rice fortification, vitamin B1, vitamin B2, vitamin B6, ultrasonication

## Abstract

As reported by the FAO, in 2022, approximately 735 million people experienced undernourishment, underscoring the critical need for effective strategies to address micronutrient deficiencies. Among these strategies, the mass fortification of staple foods, particularly rice—a dietary staple for half of the global population—has emerged as one of the most effective approaches. Conventional milling processes diminish the nutritional content of rice, necessitating the development of fortification methods to enhance its nutrient profile. This study investigates advanced fortification techniques to improve the nutritional value of rice, focusing on vitamins B1, B2, and B6, with guidelines from the US Institute of Medicine’s Dietary Reference Intakes. The results indicate that implementing ultrasonic treatments and optimal soaking conditions (60 °C for 60 min) significantly enhances the absorption of these vitamins. Effective parameters included a concentration of 1500 ppm for vitamin B1 and higher levels for vitamins B2 and B6, with a rice-to-vitamin solution ratio of 1:4. These conditions yielded an absorbed vitamin B1 content of 1050 mg/kg, bringing the fortified rice closer to meeting recommended intake levels. Given the global average daily consumption of 100 g of rice per person, this research demonstrates the feasibility of fortifying rice to address nutrient deficiencies effectively and contribute to improved dietary health worldwide. Further enhancement of vitamin B2 and B6 levels remains essential for optimal fortification, highlighting the potential of fortified rice as a sustainable solution for improving global nutrition.

## 1. Introduction

The Food and Agriculture Organization (FAO) [1] estimated that in 2022, 2.4 billion people lacked access to nutritious, safe, and sufficient food throughout the year, primarily affecting women and individuals in rural areas. Of these, approximately 735 million were experiencing undernourishment. Projections indicate that by 2030, nearly 600 million people will face chronic undernourishment, emphasizing a major hurdle in meeting the Sustainable Development Goal (SDG) of eliminating hunger. Among the primary drivers of chronic undernourishment are (i) consuming fewer calories than needed for daily energy requirements and (ii) dietary deficiencies in essential vitamins, minerals, and macronutrients. According to Peña-Rosas et al. [2], micronutrient malnutrition severely compromises health and well-being, particularly in low- and middle-income countries. Current recommended strategies for preventing and treating micronutrient deficiencies include one or a combination of the following approaches: supplementation, dietary diversification, mass food fortification, and point-of-use food fortification [2]. Food fortification is widely regarded as the most cost-effective long-term strategy for preventing micronutrient deficiencies. Governments have introduced national programs to fortify commonly consumed staple foods, such as rice, cereal flour, salt, sugar, and soy sauce. These mass fortification initiatives, often mandated by authorities, are especially effective in developing countries [3].

There have been several successful cases of supplementing staple foods to prevent nutritional deficiencies around the world. A 2005 study in *Pediatrics* found that the incidence of neural tube defects (NTDs) in infants fell by about a third in some ethnic groups after the US increased folic acid fortification of grain products in the 1990s [4]. Analyzing data from 21 surveillance systems covering around 11 million births from 1995 to 2002, the study noted significant declines in spina bifida and anencephaly cases. The prevalence of NTDs decreased significantly among Hispanic and non-Hispanic white groups, with decreases of 26% and 29% for anencephaly and 36% and 34% for spina bifida.

Costa Rica serves as a model for the successful implementation of a rice fortification program. Since the introduction of the national fortification initiative, anemia prevalence rates have significantly decreased. Between 1996 and the 2008–2009 National Survey, anemia among children aged one to six years dropped by 71.2%, with the reductions being greater in rural areas (89.6%) compared to urban areas (74.6%). Currently, national anemia prevalence ranges from 1 to 9.9%, making it no longer a public health concern. For women of childbearing age, anemia prevalence decreased by 46.8% nationally between 1996 and 2008–2009, with reductions of 54% in rural areas, 46.3% in urban areas, and 36.4% in metropolitan areas [5].

Rice (*Oryza sativa*) is a primary staple food that provides energy for half of the world’s population, particularly in the tropical regions of Asia, South America, and Africa. The nutritional value of rice depends on various factors such as its variety, climate, geography, geochemistry, farming practices, storage duration, and milling process [6]. To meet consumer preferences for refined white rice, the milling process is commonly applied [7]. However, this process often strips away most of the essential nutrients from rice grains. Additionally, washing or soaking rice multiple times further depletes its nutrients [8]. During the polishing step, essential vitamins and minerals are removed from the milled rice grains, leading to significant nutritional deficiencies and posing a serious public health concern [3]. It is therefore considered a promising strategy to enhance the nutritional content of polished rice in countries where it is a staple food to improve people’s dietary intake of nutrients [2,9,10]. Two categories of fortification methods are referred to in the literature: (a) ‘whole grain’ fortification and (b) ‘powder type’ fortification [11,12,13]. In the whole-grain approach, nutrients are applied externally or mixed with rice grains, while in the powder-type approach, nutrients are added to rice flour and then extruded to create fortified rice-based kernels. Typically, fortification is applied to a limited amount of rice kernels, which are then mixed with normal rice (i.e., at a ratio of 0.5–2%) [14].

According to the FAO [15], over 7000 plant species have been cultivated for food, yet 90% of the global dietary energy supply is sourced from just 30 species. Many other species, including numerous varieties, are classified as underutilized or neglected crops. Despite their potential to enhance food security and diversify diets, these crops are often overlooked by agricultural and nutritional researchers. The cultivation of wild crops carries significant societal benefits today, such as bolstering food security through the diversification of staple foods and offering solutions to combat climate change. This practice optimizes land use by utilizing soils unsuitable for major crops, enhances nutrition, particularly in developing regions, and creates new income opportunities for small- and medium-scale farmers [16]. Due to the aforementioned benefits, floating rice varieties have increasingly been grown in regions of the Mekong Delta of Vietnam that experience annual flooding. Floating rice varieties, which are wild rice landraces rich in essential nutrients like vitamin B1 (thiamine), vitamin B2 (riboflavin), vitamin B6 (pyridoxine), and important minerals, possess the unique capacity to thrive in flooding conditions. These varieties are cultivated without chemical fertilizers, pesticides, or insecticides, making them promising natural rice options [17]. Floating rice was used as the material for our study.

Recently, an interesting novel approach using ultrasonication has been adopted to boost vitamin absorption in rice grains using the whole-grain method [18,19]. A study by Bonto et al. [18] revealed that this combined ultrasonicated method increased vitamin B5 levels in rice by over 140% compared to the non-ultrasonicated method. Our study applied ultrasonic techniques to fortify rice through the whole-grain method. We investigated how varying factors such as the milling degree, temperature, soaking time, and rice-to-vitamin solution ratio affect the absorption of vitamins B1, B2, and B6 in floating rice grains.

## 2. Materials and Methods

### 2.1. Materials

Brown rice grains (Nang Tay Dum floating rice variety) were provided by Ngoc Troi Seed Company (Long Xuyen, Vietnam) with a moisture content of less than 14%. All rice grains were stored at 4 °C immediately upon their receipt and kept at this temperature for all studies. For experiments, brown rice grains were milled at three levels: level 3 (corresponding to a milling recovery of 85.1%), level 3.5 (73.6% milling recovery), and level 4 (68.1% milling recovery) using an MHR-1500 milling machine (Marumasu Kikai Company, Nagano, Japan). The milled rice was vacuum-sealed in light-resistant packaging and stored at 4 °C. Standard vitamin B1 with a purity of 99.98%, vitamin B2 with a purity of 99.8%, and vitamin B6 with a purity of 99.95% were purchased from the National Institute of Drug Quality Control (Ha Noi, Vietnam).

### 2.2. Sonication of Rice Grains

An aliquot of 50 g of rice grains was soaked in distilled water at a rice-to-water ratio of 1:1 (*w*/*w*) and placed in an ultrasonic bath (40 kHz, UCP-10, JeioTech, Daejeon, Republic of Korea) for 5 min without temperature control [19,20]. Afterward, the water was drained, and the rice sample was air-dried at room temperature for 12 h before being stored in vacuum-sealed packaging for further studies.

### 2.3. Fortification of Rice Grains with Vitamins B1, B2, and B6

**Figure 1:**
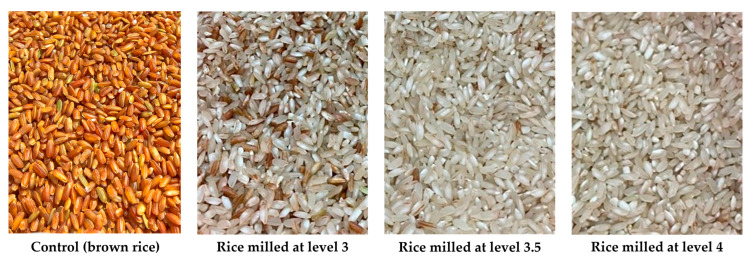
Rice grains of Nang Tay Dum floating rice variety at different degrees of milling.

First, 10 g rice grain samples, both non-sonicated (the control) and sonicated, were soaked in a solution containing vitamins B1, B2, and B6, each at a concentration of 1000 ppm, at a rice-to-vitamin solution ratio of 1:2 (*w*/*w*) for 60 min. Different milling degrees of the MHR-1500 milling machine (i.e., 3, 3.5, and 4) (Figure 1) were applied to these rice grain samples to investigate the absorption levels of vitamins B1, B2, and B6 into the milled grains. The milling degree that resulted in the best vitamin absorption was used for further tests.

For the second experiment, sonicated, milled grain samples were soaked in a solution containing vitamins B1, B2, and B6, each at a concentration of 1000 ppm, at a rice-to-vitamin solution ratio of 1:2 (*w*/*w*) at temperatures of 50, 55, 60, and 65 °C for durations of 30, 60, and 90 min to observe the vitamin absorption levels. The findings from this experiment were further applied to study the effect of different rice-to-vitamin solution ratios (i.e., 1:1, 1:2, 1:3, 1:4, and 1:5) and vitamin concentrations (i.e., 500, 1000, and 1500 ppm) on vitamin absorption levels.

**Figure 2:**
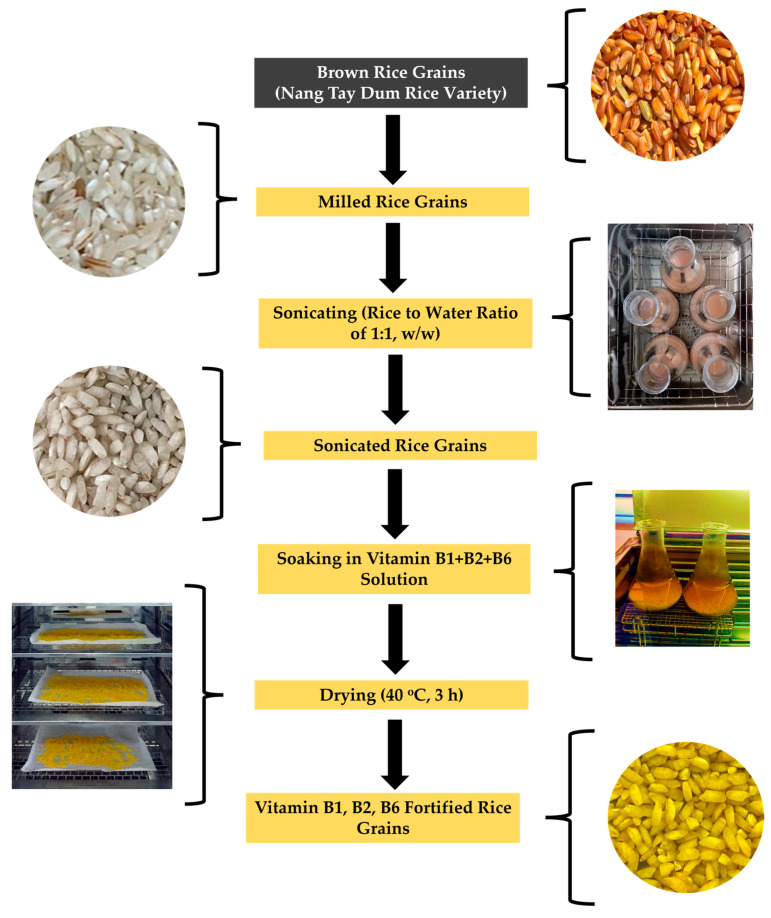
Scheme of vitamin B1, B2, and B6 fortification of the rice grains of Nang Tay Dum variety.

For all treatments, the vitamin-absorbed samples were dried in an oven at 40 °C for 3 h for further use. The scheme of vitamin B1, B2, and B6 fortification of the rice grains is presented in Figure 2.

### 2.4. Extraction Method of Vitamins B1, B2, and B6 from Rice Grains

The extraction protocol of vitamins B1, B2, and B6 from rice grains involved preparing an extraction solution by mixing 50 mL of acetonitrile with 10 mL of glacial acetic acid and then adding double-distilled water to a volume of 1000 mL. Each sample, weighed to 10 g and homogenized using a mortar and pestle, was transferred into conical flasks. A volume of 25 mL of the extraction solution was added, and the mixture was shaken in a water bath at 70 °C for 40 min. After cooling down, the sample was filtered, and the extraction solution was brought to a final volume of 50 mL. The sample was then filtered through 0.45 µm syringe filter, and aliquots of 20 µL from the solution were injected into the HPLC system using an auto-sampler.

### 2.5. Analysis of Vitamins B1, B2, and B6 by HPLC

The contents of vitamins B1, B2, and B6 absorbed in the rice grain samples were analyzed using a high-performance liquid chromatography (HPLC) system equipped with a UV-Vis detector (Shimadzu HPLC Prominence-i system, Shimadzu Corporation, Kyoto, Japan). Chromatographic separation was performed on an Ecosil C18 column (250 mm × 4.6 mm × 5 µm) (Guangzhou Lubex Scientific Instrument Co., Ltd., Guangzhou, China). Elution was carried out at a constant flow rate of 1.0 mL/min with an injection volume of 10 µL and a total run time of 35 min per sample injection. The mobile-phase buffer comprised 1.08 g of sodium hexanesulfonate, 1.36 g of potassium dihydrogen phosphate, 940 mL of HPLC water, and 5 mL of triethylamine and methanol mixed at a ratio of 96:4. Detection was monitored at 210 nm for vitamins B1 and B6, and at 266 nm for vitamin B2. The column temperature was maintained at ambient conditions.

### 2.6. Data Analysis

Analysis of variance (ANOVA) was applied to evaluate the differences between the means of multiple groups under various treatments, followed by Tukey’s test using GraphPad Prism version 9.0 (GraphPad Software Inc., San Diego, CA, USA). The data are presented as the mean of triplicate measurements ± standard deviation (±SD), with a statistical significance of *p* < 0.05.

Many empirical polynomial models describing the relationship between the predicted response (i.e., absorbed vitamin B1, B2, and B6 levels in this case) and the independent variables (i.e., temperature, time, or vitamin concentration and rice-to-vitamin solution ratio) have been formulated [21,22,23,24]. Among these, the second-order polynomial model for two factors is addressed in this study (Equation (1)).

(1) $$Y=\beta_{0}+\beta_{1}X_{1}+\beta_{2}X_{2}+\beta_{11}X_{1}^{2}+\beta_{22}X_{2}^{2}+\beta_{12}X_{1}X_{2},$$

where *Y* is the predicted response; *β*_0_ is the constant; *β*_1_, *β*_2_, *β*_11_, *β*_22_, and *β*_12_ are the unknown parameters of the variables for the linear, quadratic, and interaction terms, respectively; and *X*_1_ and *X*_2_ are the independent variables.

## 3. Results and Discussion

### 3.1. Nutritional Composition of Brown Rice Grains (Nang Tay Dum Floating Rice Variety)

The nutritional composition of floating brown rice grains of the Nang Tay Dum variety was analyzed and is presented in Table 1. As shown in Table 1, the moisture content of the Nang Tay Dum floating rice grains is 11.67%, which is within the suitable range for rice storage and distribution. The protein content of this rice variety is 9.20 g/100 g, which is higher than that of brown rice grains from an early, long grain, non-glutinous, non-waxy rice (*Indica* rice) originating from Southern China (8.59 g/100 g) as reported by Li et al. [25]. It is also higher than the protein content of brown rice grains from India (7.1–8.3 g/100 g) as reported by Pandey et al. [26] and higher than that of red rice grains from different genotypes originating from Brazil (6.9–7.8 g/100 g as reported by Ascheri et al. [27] and 7.89 g/100 g as reported by Müller et al. [28]). In contrast, the protein content is slightly lower than that of the red rice grains of the Huyet Rong variety originating from Vietnam (9.77 ± 0.06 g/100 g) as reported by Duong and Nguyen [29].

The lipid content of the floating rice grains is 1.79 g/100 g, which is comparable to that of red rice grains (1.6–2.2 g/100 g) originating from Brazil, as reported by Ascheri et al. [27]. The carbohydrate content of the rice grains is 73.3 g/100 g, similar to that of brown rice grains (73–76 g/100 g) as reported by Saleh et al. [30]. This carbohydrate content falls within the range of 73 to 83 g/100 g for brown rice varieties originating from India, as reported by Pandey et al. [26], and the range of 71.31 to 84.75 g/100 g for brown rice varieties originating from South India, as reported by Rathna Priya et al. [31]. The vitamin B1 content in the floating rice grains of this study was 0.413 mg/100 g, which is consistent with the range reported by Saleh et al. (0.29–0.61 mg/100 g) [30]. The vitamin B2 content was 0.154 mg/100 g, compared to 0.04 to 0.14 mg/100 g for brown rice grains as reported by Saleh et al. [30]. The vitamin B6 content was 0.112 mg/100 g, which is comparable to that in white rice (0.103 mg/100 g) but lower than that in brown rice (0.294 mg/100 g) as reported by Zahra and Jabeen [32].

### 3.2. Effect of Milling Degree on the Absorption of Vitamins B1, B2, and B6 in Nang Tay Dum Floating Rice Grains

**Figure 3:**
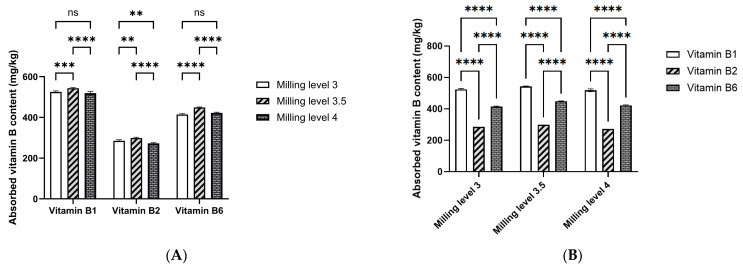
Effect of milling degree on the absorption of vitamins B1, B2, and B6 in Nang Tay Dum floating rice grains. The data were calculated based on the average ± standard deviation of triple repetitions, and the data analyses were based on one-way ANOVA (**** indicates *p* < 0.0001, *** *p* < 0.001, ** *p* < 0.01, and ns means no significant difference). (**A**) Test used to evaluate the difference between the means of vitamin absorption at different milling levels; (**B**) Test used to evaluate the difference between the means of vitamin absorption for different types of vitamins.

The brown rice grains of the Nang Tay Dum variety were first milled, then sonicated, soaked in a solution containing 1000 ppm each of vitamins B1, B2, and B6, and finally dried under the conditions specified in Section 2.3. The effect of the milling degree on the absorption of vitamins B1, B2, and B6 in the rice grains was investigated and is illustrated in Figure 3. As shown, the degree of milling significantly affects vitamin absorption in the rice grains. Milling impacts the removal of both major and minor nutrients, influencing the absorption of vitamins and other essential elements such as iron, zinc, thiamine, riboflavin, and various minerals [33,34,35,36].

The absorption of vitamin B1 in rice grains with a milling degree of 3.5 reached a peak level of 541.0 ± 3.6 mg/kg, significantly higher than that at a milling degree of 4, but not significantly different from the level at a milling degree of 3. Similarly, the absorption of vitamin B6 at a milling degree of 3.5 was 452.0 ± 7.9 mg/kg, significantly higher than at milling degrees of 3 and 4. In contrast, the absorption of vitamin B2 across the three milling degrees was relatively low, ranging from 279.0 to 293.3 mg/kg.

As depicted in Figure 3A,B, the type of vitamin significantly affects its absorption in rice grains. The mean absorption levels observed were 533.9 ± 4.2 mg/kg for vitamin B1, 435.9 ± 14.4 mg/kg for vitamin B6, and 286.1 ± 7.1 mg/kg for vitamin B2.

These findings align with data reported by Reddy et al. [37] and Bonto et al. [18], who investigated vitamin B5 fortification in sonicated rice grains at a milling level corresponding to 27% (level 3.5 in our study). In their research, vitamin B5 absorption levels ranged from 450 to 600 mg/kg, depending on the specific type of vitamin and its absorption efficiency.

### 3.3. Effect of Soaking Time and Temperature on the Absorption of Vitamins B1, B2, and B6 in Rice Grains

Brown rice grains, milled to a level of 3.5 (with a milling recovery of 73.6%), were first sonicated and then soaked in a solution with a rice-to-solution ratio of 1:2 (*w*/*w*) containing vitamins B1, B2, and B6, each at a concentration of 1000 ppm. The effects of varying soaking times (30 to 90 min) and temperatures (50 to 65 °C) on the absorption capacity of these vitamins in the milled rice grains are presented in Table 2.

As observed, increasing the soaking time from 30 to 60 min resulted in a rise in the vitamin B1 content absorbed into the rice grains, reaching its peak at 60 min. This peak absorption was significantly higher compared to the absorption at 30 min. However, extending the soaking time to 90 min significantly decreased the vitamin B1 content compared to that at the 60 min mark. Across different soaking temperatures, the vitamin B1 content absorbed was high and consistent between 50 and 60 °C but decreased significantly at 65 °C compared to 60 °C [18,38,39].

For vitamin B2, absorption into the rice grains increased significantly from 30 to 60 min, reaching a higher level at 60 min. However, extending the soaking time to 90 min did not result in significant differences compared to the 30 and 60 min soaking periods. Regarding temperature, the vitamin B2 content was significantly higher at 55 and 60 °C compared to 50 and 65 °C.

The absorption of vitamin B6 showed no significant differences with changes in soaking time from 30 to 90 min. However, the content absorbed varied significantly with temperature changes, peaking at 60 °C.

According to Bonto et al. [18], the uptake of vitamin B5 into sonicated TH82 rice reached an equilibrium after 60 min of soaking. Beyond one hour, the absorbed vitamin B5 concentration leveled off, suggesting equilibrium or supersaturation. Bello et al. [40], who studied the hydration of rough rice grains in hot water at temperatures ranging from 25 to 90 °C, concluded that concurrent processes related to water diffusion and starch gelatinization occur during rice soaking. According to these authors, 60 °C serves as a critical temperature point. This break temperature aligns with the experimentally determined gelatinization temperature of rice grains. Below 60 °C, water diffusion is the controlling factor, while above 60 °C, the reaction of water with starch (starch gelatinization) becomes dominant.

Additionally, vitamin B2 has unique characteristics among the B vitamins, being sensitive to temperature and light, and exhibiting a distinct yellow color [41]. Consequently, the vitamin B2 absorbed into the rice grains is less stable compared to vitamins B1 and B6, resulting in about half the absorption value compared to that of vitamin B1. This finding is consistent with data reported in previous studies [11,13,42].

### 3.4. Modeling the Combined Effect of Soaking Time and Temperature on the Absorption of Vitamins B1, B2, and B6 in Rice Grains

By fitting Equation (1), where *X*_1_ represents the soaking time variable and *X*_2_ represents the temperature variable, to the experimental data, the model parameters were estimated using a nonlinear regression analysis (proc NLIN, SAS 9.1, SAS Institute Inc., Cary, NC, USA). However, for the case of vitamin B1 absorption, the term *β*_0_ was found to be redundant, as indicated by a large standard error (>100%). Consequently, this term was omitted, and a reduced version of Equation (1), i.e., Equation (2), was used. The model parameters estimated based on Equations (1) and (2) are presented in Table 3.

(2) $$Y=\beta_{1}X_{1}+\beta_{2}X_{2}+\beta_{11}X_{1}^{2}+\beta_{22}X_{2}^{2}+\beta_{12}X_{1}X_{2},$$

**Figure 4:**
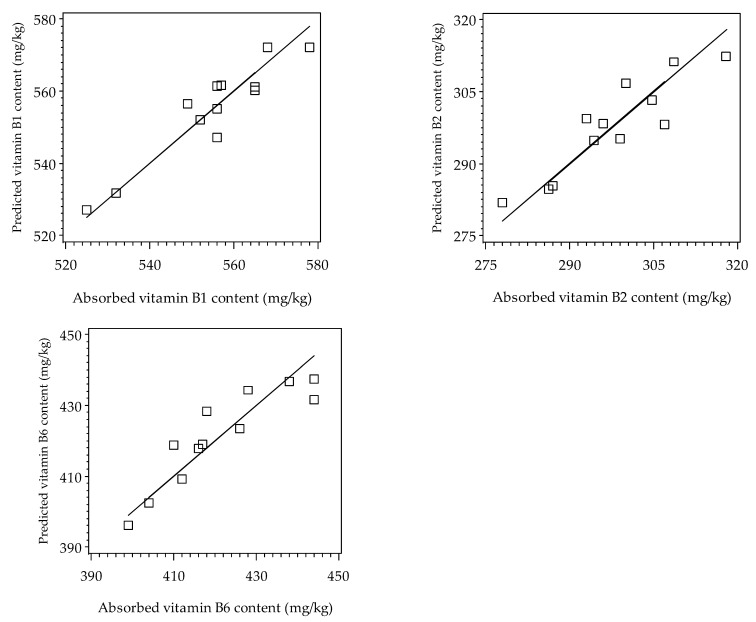
Correlation between the experimental vitamin contents and the predicted vitamin contents for the absorption of vitamins B1, B2, and B6 into the rice grains according to the second-degree polynomial models (Equations (1) and (2)).

For Equations (1) and (2), no tendency was observed when plotting residuals (the differences between the experimental and predicted absorbed contents of vitamins B1, B2, and B6, respectively) against the soaking time, temperature, experimental vitamin contents, and predicted vitamin contents. Additionally, parity plots comparing the predicted vitamin contents from Equations (1) and (2) with the experimental vitamin contents were created for vitamins B1, B2, and B6 (Figure 4). The deviation from the bisector in these plots indicates model inaccuracy. The smaller the difference between the experimental and predicted vitamin contents, the more accurate the models. Good agreement between the predicted and experimental vitamin contents was observed for the models, indicating their success [21].

**Figure 5:**
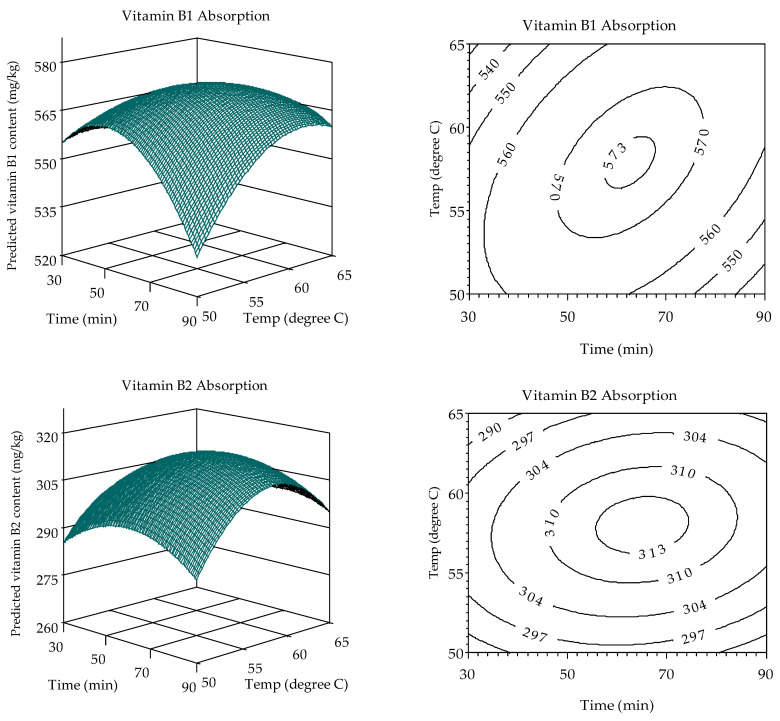

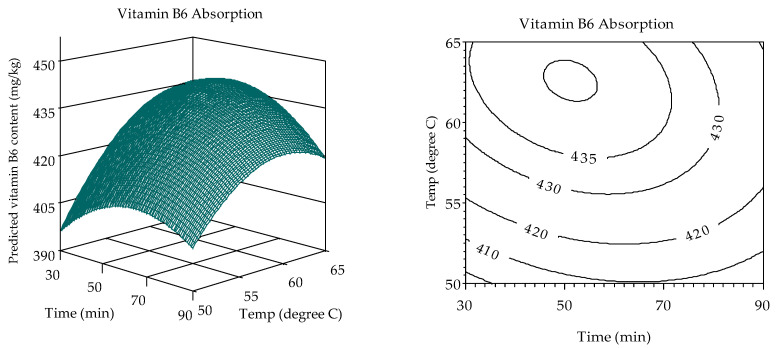
Three-dimensional and isorate contour plots depicting the absorption capacity of vitamins B1, B2, and B6 in floating rice grains as a function of soaking time and temperature. (**Left**): 3D plots; (**Right**): isorate contour plots based on reconstructed second-degree polynomial models (Equations (1) and (2)).

By applying the model parameters from Table 3 to Equations (1) and (2), simulations were conducted to determine the soaking time and temperature combinations that influence the absorption of vitamins B1, B2, and B6 in the rice grains. The results are illustrated in three-dimensional and isorate contour plots (Figure 5).

### 3.5. Effects of Vitamin Concentration and Rice-to-Solution Ratio on the Absorption of Vitamins B1, B2, and B6 in Rice Grains

**Figure 6:**
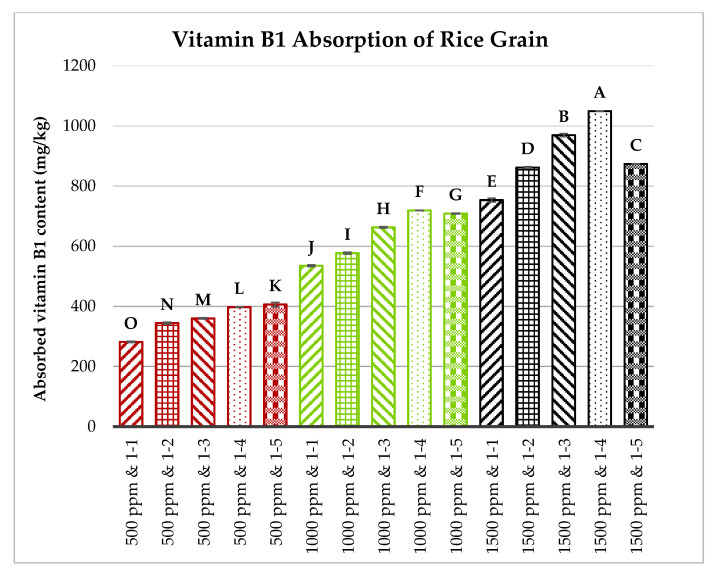
Effects of vitamin B1 concentration and rice-to-vitamin solution ratio on the absorption capacity of vitamin B1 in floating rice grains. Data were calculated based on the mean of three repetitions, using a one-way ANOVA. Means with the same letter are not significantly different.

**Figure 7:**
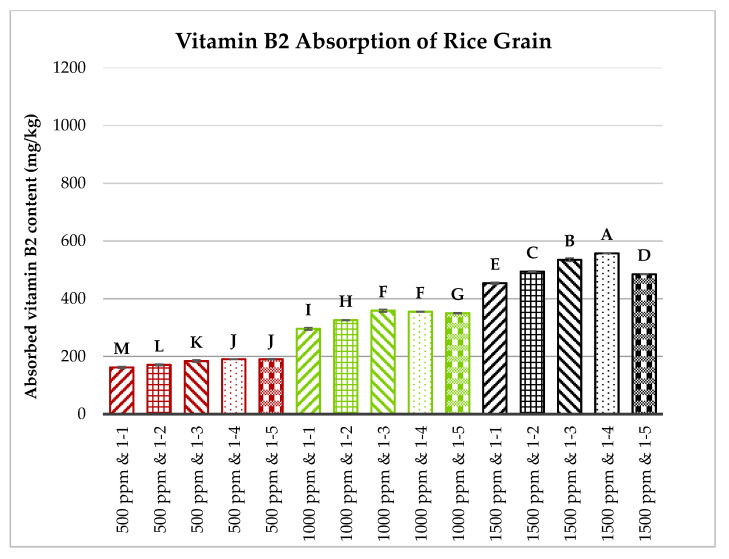
Effects of vitamin B2 concentration and rice-to-vitamin solution ratio on the absorption capacity of vitamin B2 in floating rice grains. Data were calculated based on the mean of three repetitions, using a one-way ANOVA. Means with the same letter are not significantly different.

**Figure 8:**
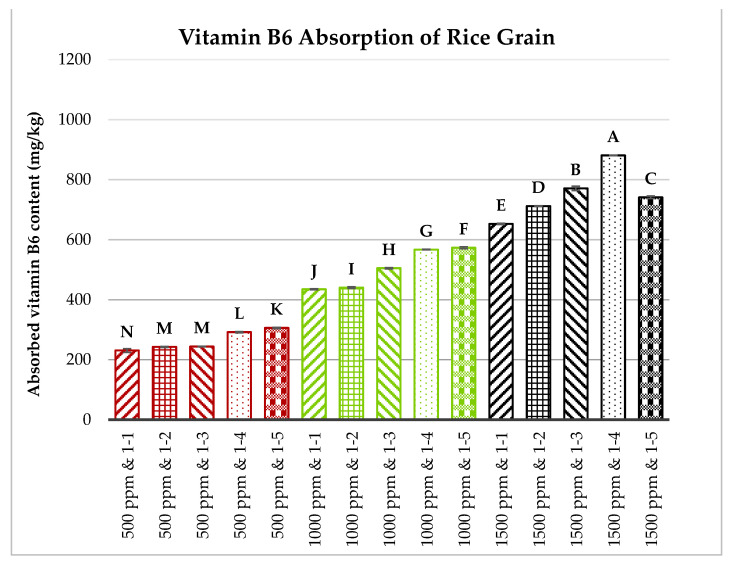
Effects of vitamin B6 concentration and rice-to-vitamin solution ratio on the absorption capacity of vitamin B6 in floating rice grains. Data were calculated based on the mean of three repetitions, using a one-way ANOVA. Means with the same letter are not significantly different.

Brown rice grains, milled to a level of 3.5, were first sonicated and then soaked in solutions containing vitamins B1, B2, and B6, each at concentrations of 500, 1000, or 1500 ppm. The rice-to-vitamin solution ratios varied from 1:1 to 1:5 (*w*/*w*). The soaking process lasted 60 min at 60 °C. The absorption capacities of vitamins B1, B2, and B6 by the rice grains were measured and are illustrated in Figure 6, Figure 7 and Figure 8.

Figure 6, Figure 7 and Figure 8 show that the concentration of vitamins B1, B2, and B6 in the soaking solutions significantly affects their absorption by the rice grains. As the vitamin concentration increased from 500 to 1500 ppm, the rice grains absorbed more of these vitamins. This trend aligns with the findings of Kyritsi et al. [13] on vitamin B group fortification in white rice grains. Additionally, increasing the rice-to-vitamin solution ratio from 1:1 to 1:4 enhanced the absorption capacity, peaking at a ratio of 1:4 for most treatments, except for vitamin B1 at 500 ppm and vitamin B6 at 500 and 1000 ppm. However, at 1500 ppm, the absorption significantly decreased when the ratio increased to 1:5 compared to 1:4 for all three vitamins.

**Figure 9:**
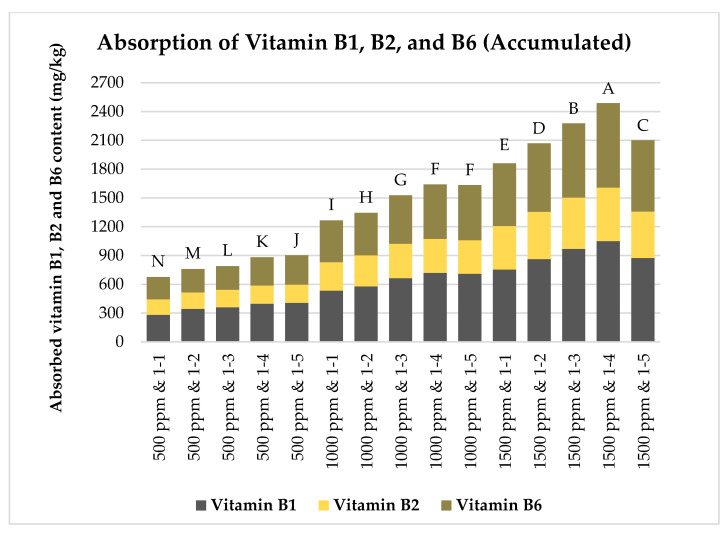
Effects of vitamin B1, B2, and B6 concentrations and rice-to-vitamin solution ratios on the accumulated absorption capacities of vitamins in floating rice grains. Data were calculated based on the mean of three repetitions, using a one-way ANOVA. Means with the same letter are not significantly different.

The accumulated absorption capacities of vitamins B1, B2, and B6 for each combination of vitamin concentration and rice-to-vitamin solution ratio were determined and are plotted as a function of the vitamin concentration and solution ratio, as shown in Figure 9. Figure 9 demonstrates a significant relationship between the concentration of vitamins in the soaking solutions and their accumulated absorption by rice grains. As the vitamin concentration increased from 500 to 1500 ppm, the rice grains showed an increased accumulative absorption of these vitamins, consistent with the findings shown in Figure 6, Figure 7 and Figure 8 in this study. Additionally, increasing the rice-to-vitamin solution ratio from 1:1 to 1:4 generally boosted the absorption capacity, peaking at 1:4 for most treatments, except at 500 ppm. However, at 1500 ppm, the accumulative absorption notably decreased when the ratio shifted from 1:4 to 1:5.

As published by the Institute of Medicine (US), the Dietary Reference Intakes (DRIs) for vitamin B1, vitamin B2, and vitamin B6 for adults are delineated as follows. For vitamin B1, the recommended daily intake is 1.2 mg for men and 1.1 mg for women. Vitamin B2 intake should be 1.3 mg per day for men and 1.1 mg per day for women. Regarding vitamin B6, individuals aged 19–50 years require 1.3 mg per day, while men aged 51 years and older need 1.7 mg per day, and women of the same age group require 1.5 mg per day. These DRIs represent the average daily intake levels deemed sufficient to meet the nutritional requirements of nearly all (97–98%) healthy individuals within specific age and gender categories [43].

The average amount of rice consumed per person per day varies significantly by region and dietary habits. In Asia, where rice is a staple food, the average consumption can be quite high, with countries like Bangladesh, Vietnam, and Indonesia seeing a daily consumption per person of around 200 to 300 g (uncooked). In other parts of the world, such as the United States or Europe, the daily rice consumption per person is much lower, averaging around 30 to 50 g (uncooked). Taking these variations into account, a global average might be approximately 100 g (uncooked) per person per day [44].

Assuming a daily rice consumption of 100 g per person, and a vitamin B1, B2, and B6 content in the rice premix (vitamin-fortified rice) of 1 mg/g (i.e., 1000 mg/kg), mixing the premix with non-fortified rice at a ratio of 1.2:100 to 1.3:100 (*w*/*w*) [14] will produce fortified rice that meets the Dietary Reference Intake for vitamins B1, B2, and B6. In this study, the best absorbed vitamin B1 content in the rice premix is 1050 mg/kg, making it ready for mixing. However, the best vitamin B2 and B6 contents are lower, at 557 mg/kg and 881 mg/kg, respectively, and need to be increased to approximately 1000 mg/kg.

**Figure 10:**
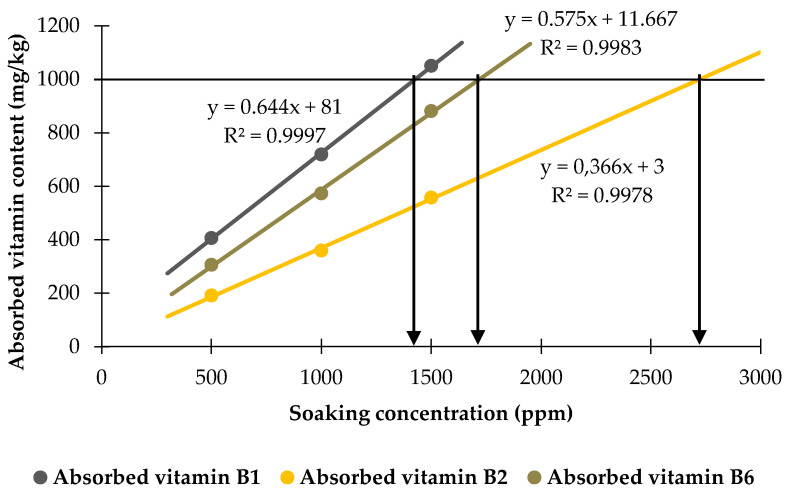
Linear relationship between the absorbed vitamin content and the vitamin concentration in the soaking solutions.

The highest absorption capacity of these vitamins, at the rice-to-vitamin solution ratio of 1:4, was plotted against the vitamin concentration and is presented in Figure 10. It was observed that the increase in absorption by rice grains follows a linear relationship with the vitamin concentration in the soaking solutions, showing a strong correlation. These data serve as a valuable reference for predicting the required vitamin concentration in soaking solutions to achieve a targeted absorbed vitamin content.

## 4. Conclusions

This study demonstrates the potential of advanced fortification techniques to improve the nutritional value of rice, a staple food for many consumers worldwide. The Dietary Reference Intakes for vitamins B1, B2, and B6 set by the Institute of Medicine (US) are essential guidelines for adequate daily intake. Given the high rice consumption in regions such as Asia, Africa, South America, and parts of the Middle East, fortifying rice to meet these DRIs can effectively address nutrient deficiencies.

Using ultrasonic treatment and optimal soaking conditions at 60 °C for 60 min significantly enhanced the absorption of vitamins B1, B2, and B6. The most effective parameters were 1500 ppm for vitamin B1, higher concentrations for vitamins B2 and B6, and a rice-to-vitamin solution ratio of 1:4. These conditions brought the fortified rice close to meeting the DRIs for these vitamins.

Given a global average daily rice consumption of 100 g per person, it is feasible to achieve recommended vitamin intake levels by fortifying rice at appropriate concentrations. The study achieved an absorbed vitamin B1 content of 1050 mg/kg in the rice premix, ready for blending, while the vitamin B2 and B6 contents were lower, indicating a need for further enhancement.

## Figures and Tables

**Table 1:** Nutritional composition of brown rice grains (Nang Tay Dum floating rice variety).

Nutritional Composition ^1^	Unit	Content
Moisture	%	11.67 ± 0.01
Crude protein	g/100 g	9.20 ± 0.07
Lipid	g/100 g	1.79 ± 0.03
Carbohydrate	g/100 g	73.3
Vitamin B1	mg/100 g	0.413
Vitamin B2	mg/100 g	0.154
Vitamin B6	mg/100 g	0.112

^1^ Data are indicated as mean ± SD (*n* = 3).

**Table 2:** Absorbed vitamin content in rice grains at different soaking time and temperature combinations (mg/kg).

	Mean of Absorbed Vitamin B1 Content ^2^			
	Soaking Time (min)			
Temperature (°C)	30	60	90	Group mean
50	556.3 ± 0.9	565.0 ± 0.1	532.0 ± 0.3	551.1 ± 15.4 ^AB^
55	549.0 ± 0.1	568.0 ± 0.8	552.3 ± 0.6	556.4 ± 9.4 ^AB^
60	556.0 ± 0.1	577.8 ± 1.0	557.0 ± 0.6	563.6 ± 11.2 ^A^
65	525.0 ± 0.6	556.0 ± 0.6	565.0 ± 0.4	548.7 ± 18.3 ^B^
Group mean	546.6 ± 14.8 ^b^	566.7 ± 9.0 ^a^	551.6 ± 14.1 ^b^	555.0 ± 10.5
	Mean of absorbed vitamin B2 content ^2^			
	Soaking Time (min)			
Temperature (°C)	30	60	90	Group mean
50	287.0 ± 0.5	294.3 ± 0.7	286.3 ± 0.4	289.2 ± 5.3 ^B^
55	293.0 ± 0.4	308.7 ± 0.2	304.7 ± 0.3	302.1 ± 7.5 ^A^
60	307.0 ± 0.6	318.0 ± 0.1	300.0 ± 0.4	308.3 ± 8.1 ^A^
65	278.0 ± 0.3	296.0 ± 0.6	299.0 ± 0.6	291.0 ± 9.9 ^B^
Group mean	291.3 ± 12.2 ^b^	304.3 ± 11.2 ^a^	297.5 ± 7.8 ^ab^	297.7 ± 6.5
	Mean of Absorbed Vitamin B6 Content ^2^			
	Soaking Time (min)			
Temperature (°C)	30	60	90	Group mean
50	399.3 ± 0.5	412.0 ± 0.5	404.0 ± 0.5	405.1 ± 7.9 ^D^
55	410.0 ± 0.6	418.0 ± 0.2	416.0 ± 0.5	414.7 ± 4.8 ^C^
60	444.0 ± 0.8	444.0 ± 0.6	426.0 ± 0.7	438.0 ± 9.9 ^A^
65	428.0 ± 0.5	438.0 ± 0.2	417.0 ± 0.2	427.7 ± 9.3 ^B^
Group mean	420.3 ± 19.7 ^a^	428.0 ± 15.4 ^a^	415.8 ± 9.0 ^a^	421.4 ± 6.2

^2^ Data are indicated as mean ± SD (*n* = 3) of each combined temperature and soaking time. Means within the last column or row followed by the same capital letter or lowercase letter are not significantly different at *p* ≤ 0.05.

**Table 3:** Estimated model parameters for the time–temperature absorption of vitamins B1, B2, and B6 into the rice grains based on the second-degree polynomial model.

Parameter	Vitamin B1 (Equation (2))	Vitamin B2 (Equation (1))	Vitamin B6 (Equation (1))
*β*_0_	-	−692.7 ± 247.4	−450.3 ± 337.5
*β*_1_ (*X*_1_: time)	1.1613 ± 0.9069	0.5315 ± 0.9377	2.6383 ± 1.2792
*β*_2_ (*X*_2_: temp)	21.0620 ± 0.9789	34.1107 ± 8.5372	26.2667 ± 11.6456
*β*_11_ (*X*_1_^2^: time^2^)	−0.0198 ± 0.0043	−0.0110 ± 0.0044	−0.0111 ± 0.0059
*β*_22_ (*X*_2_^2^: temp^2^)	−0.2159 ± 0.0153	−0.3027 ± 0.0738	−0.2000 ± 0.1007
*β*_12_ (*X*_1_ × *X*_2_: time × temp)	0.0629 ± 0.0130	0.0155 ± 0.0135	−0.0240 ± 0.0184
Corrected *R*^2^	0.99	0.81	0.80
Standard Deviation (SD)	6.29	1.65	2.25

## Data Availability

The original contributions presented in the study are included in the article, further inquiries can be directed to the corresponding author.
